# Advancing ADAS Perception: A Sensor-Parameterized Implementation of the GM-PHD Filter

**DOI:** 10.3390/s24082436

**Published:** 2024-04-11

**Authors:** Christian Bader, Volker Schwieger

**Affiliations:** 1 Institute of Engineering Geodesy, University of Stuttgart, Geschwister-Scholl-Str. 24D, 70174 Stuttgart, Germany; volker.schwieger@iigs.uni-stuttgart.de; 2 Daimler Truck AG, Fasanenweg 10, 70771 Leinfelden-Echterdingen, Germany

**Keywords:** GM-PHD filter, multi-object tracking, sensor fusion

## Abstract

Modern vehicles equipped with Advanced Driver Assistance Systems (ADAS) rely heavily on sensor fusion to achieve a comprehensive understanding of their surrounding environment. Traditionally, the Kalman Filter (KF) has been a popular choice for this purpose, necessitating complex data association and track management to ensure accurate results. To address errors introduced by these processes, the application of the Gaussian Mixture Probability Hypothesis Density (GM-PHD) filter is a good choice. This alternative filter implicitly handles the association and appearance/disappearance of tracks. The approach presented here allows for the replacement of KF frameworks in many applications while achieving runtimes below 1 ms on the test system. The key innovations lie in the utilization of sensor-based parameter models to implicitly handle varying Fields of View (FoV) and sensing capabilities. These models represent sensor-specific properties such as detection probability and clutter density across the state space. Additionally, we introduce a method for propagating additional track properties such as classification with the GM-PHD filter, further contributing to its versatility and applicability. The proposed GM-PHD filter approach surpasses a KF approach on the KITTI dataset and another custom dataset. The mean OSPA^(2)^ error could be reduced from 1.56 (KF approach) to 1.40 (GM-PHD approach), showcasing its potential in ADAS perception.

## 1. Introduction

Modern vehicles are equipped with a variety of Advanced Driver Assistance Systems (ADAS) functions, such as advanced emergency braking assistance, blind spot information system, and adaptive cruise control. These functions actively support the driver and require a reliable environment perception, typically on the object track level. Current-generation vehicles with Level 2 systems fuse and track object detections from multiple sensors with different sensing domains and mounting positions to increase the overall system’s Field of View (FoV), performance, and robustness.

This multi-sensor multi-object tracking problem can be solved by a variety of approaches. Approaches that estimate full trajectories by taking all measurements into account at once, such as shadowing filters [1], are able to achieve good tracking results; however, these are typically not capable of processing the data sequentially, which is required by most online applications, where at each time only the measurements of the current time-step are known. Particle filters are one type of online-capable approach that processes measurements recursively. They have particular strengths in representing the uncertainties of nonlinear systems and non-Gaussian noise, but require a large number of particles to do this accurately, especially for high-dimensional systems. Although real-time-capable implementations are possible, as shown by [2] using Rao–Blackwellization, the authors still estimated the computational speed to be slower compared to a Kalman filter by a factor of 100 with $10^{3}$ particles and a state vector of dimension $n_{x}\approx 5$. Because this paper is interested in solutions with computational requirements in the range of the Kalman filter and is less focused on highly nonlinear and non-Gaussian applications, the particle filter is not considered a viable choice.

A popular and computationally efficient solution to the multi-sensor multi-object tracking problem is the use of a Kalman Filter (KF), such as the examples in [3,4,5,6,7,8]. The states of each object are estimated by a separate KF [9], which essentially makes it an extension of a single target filtering approach. Therefore, such approaches require a data association scheme that matches new measurements to the already tracked objects and a track management scheme to handle the appearance and disappearance of tracks. Errors such as incorrect assignment during data association can occur during these two supporting steps. Such a situation can then lead to incorrect estimation of the number of objects and their states.

In contrast, the family of Random Finite Set (RFS) filters estimates both the cardinality (the number of objects) and their states. Therefore, they offer true multi-target filtering without data association along with complex track management. The Probability Hypothesis Density (PHD) filter [10] estimates the probability hypothesis density (or intensity function) of a random set variable. The Gaussian Mixture Probability Hypothesis Density (GM-PHD) filter [11] is an efficient Gaussian implementation of the PHD filter. This paper shows an approach that makes this filter an alternative to KF with a performance advantage that is computationally lightweight and suitable for real-time applications. A key aspect of the presented approach is that it is designed in such a way that a Kalman filter-based approach can be replaced without much effort. Therefore, it is applicable to systems with partially overlapping or non-overlapping sensor FoVs and asynchronous sensor updates, which are common in real-world setups currently covered by KF approaches.

The standard GM-PHD filter assumes the use of a single sensor. Next to the single sensor PHD filter, Mahler introduced the inter-corrector PHD [10] as a multi-sensor solution. This was achieved using multiple single-sensor Bayesian filters [12]. Improvements for multi-sensor applications of the PHD filter followed in [13] along with Gaussian mixture implementations and developments in [14,15]. However, these approaches assume sensor systems with completely overlapping FoVs and synchronous measurements, which is not the case in current-generation Level 2+ systems. In general, many GM-PHD-based fusion approaches use multiple single sensor filters together with a combination rule such as the Generalized Covariance Intersection (GCI) for fusion. These approaches are usually not suitable for partial/non-overlapping sensor FoVs. To overcome this shortage, ref. [16] presented a collaborative GM-PHD filter that fuses the intensity functions of multiple filter sources using Covariance Intersection on nearby Gaussian components only.

In [17], a comparison of two approaches to asynchronous radar sensor fusion with the PHD filter was presented. Sequence process PHD (SP-PHD) fusion fuses measurements of multiple sensors sequentially using a global filter, while fixed nodes PHD fusion uses separate sensor PHD filters that are fused using the GCI. This was followed by [18], where distributed PHD fusion approaches were shown next to the SP-PHD fusion for asynchronous sensor setups. However, these approaches have not been described for partially overlapping FoVs. Overall, there are only a very few publications focusing on real-world automotive applications, where asynchronous sensor setups and partially/non-overlapping sensor FoVs are common.

Most publications have assumed constant values for sensor-specific parameters such as clutter density and detection probability. However, this is not the case for real-world sensors, and can lead to tracking errors in the case of partial/non-overlapping sensor FoVs. This paper shows that the application of sensor-specific models for these parameters can solve multiple problems of GM-PHD filters in real-world applications. In [19], this was addressed by differentiating the detection probability between inside and outside the FoV [16]. In addition [20], introduced occlusion models for those areas where tracks are not detectable. Ref. [21] proposed a detection probability model for sonar applications, taking the object distance into account. This paper proposes a more versatile model for the detection probability with distance dependency that fits the capabilities of automotive sensors. Compared to previous publications, the clutter density is modeled in a similar way.

Because many systems fuse additional track attributes such as the classification in addition to the track states, this paper presents a new approach to handle the propagation of additional attributes with the GM-PHD filter. In addition, an adaptive birth model and a gating process are proposed to increase computational efficiency.

The presented approach uses an SP-PHD scheme for asynchronous sensor measurements in a way that can replace typical KF implementations. This enables a direct performance comparison of the proposed GM-PHD approach to a KF approach on both the KITTI dataset [22] and a custom dataset with multiple radars and a camera. In the evaluation, the proposed GM-PHD approach substitutes for the KF approach, with as many parameters as possible remaining the same in order to ensure robust comparability. By testing on two real-world datasets and considering multiple sensor combinations, a statement can be made about its suitability for real systems, which has not been possible to date.

## 2. The GM-PHD Filter

RFS filters estimate a set of multiple-target states; as such, they cover both the state estimation and the estimation of the number of states with their birth and death. Therefore, no explicit data association between individual measurements and tracks is required, as the state set is estimated as a whole.

The PHD filter [10] approximates the exact multi-target filter by only propagating the first-order statistical moment (PHD) of an RFS. The PHD is also called the intensity function. Under the assumption of a Poisson RFS, there is a closed-form analytic solution for the prediction and update step, as both the cardinality distribution and the spatial distribution are defined by the PHD.

The GM-PHD filter uses a Gaussian mixture to represent the PHD $v_{k-1}\left(x\right)$ of an object state x at time step k−1, which has the advantage of fast computation. Because the first-order statistical moment of an RFS is estimated, the PHD $v(x_{0})$ can be interpreted as the density of the expected number of targets at $x_{0}$. The PHD is always represented as a sum of $J_{k-1}$ weighted Gaussians with weight $w_{k-1}^{\left(i\right)}$, mean $m_{k-1}^{\left(i\right)}$, and covariance $P_{k-1}^{\left(i\right)}$ [11]:

(1) $$v_{k-1}\left(x\right)=\sum_{i=1}^{J_{k-1}}w_{k-1}^{\left(i\right)}N\left(x;m_{k-1}^{\left(i\right)},P_{k-1}^{\left(i\right)}\right).$$

### 2.1. GM-PHD Prediction

Similar to the Kalman filter, the GM-PHD filter is divided into prediction and update. However, in practical implementations the additional computation steps prune and merge are used to keep the number of Gaussian components at a low number, as shown by [23,24]. The GM-PHD predicted intensity $v_{k|k-1}\left(x\right)$ is calculated by combining the prediction $v_{S,k|k-1}\left(x\right)$ with the spawn intensity $v_{\beta ,k|k-1}\left(x\right)$ and birth intensity $\gamma_{k}\left(x\right)$ [11]:

(2) $$v_{k|k-1}\left(x\right)=v_{S,k|k-1}\left(x\right)+v_{\beta ,k|k-1}\left(x\right)+\gamma_{k}.$$

The spawn intensity models the spawning of new targets from existing ones, while the birth intensity models the birth of new targets; both are represented as a Gaussian mixture. The developed model for the birth intensity is shown in Section 3.4.2, while the spawn intensity is not used here. The prediction $v_{S,k|k-1}\left(x\right)$ is calculated by

(3) $$\begin{array}{cc} v_{S,k|k-1}\left(x\right) & =p_{S,k}\sum_{j=1}^{J_{k-1}}w_{k-1}^{\left(j\right)}N\left(x;m_{S,k|k-1}^{\left(j\right)},P_{S,k|k-1}^{\left(j\right)}\right), \end{array}$$

(4) $$\begin{array}{cc} m_{S,k|k-1}^{\left(j\right)} & =A_{k-1}m_{k-1}^{\left(j\right)}, \end{array}$$

(5) $$\begin{array}{cc} P_{S,k|k-1}^{\left(j\right)} & =Q_{k-1}+A_{k-1}P_{k-1}^{\left(j\right)}A_{k-1}^{T}, \end{array}$$

using the survival probability $p_{S,k}$ [11]. The means and covariances are predicted using the state transition matrix $A_{k-1}$ and process noise covariance matrix $Q_{k-1}$. Similar to the extended Kalman filter, an extended Kalman PHD filter equation set is described in [11], which allows nonlinear process models.

### 2.2. GM-PHD Update

After the prediction step, the predicted intensity $v_{k|k-1}\left(x\right)$ for time *k* is represented by the Gaussian mixture

(6) $$v_{k|k-1}\left(x\right)=\sum_{i=1}^{J_{k|k-1}}w_{k|k-1}^{\left(i\right)}N\left(x;m_{k|k-1}^{\left(i\right)},P_{k|k-1}^{\left(i\right)}\right).$$

The GM-PHD update step then calculates the corrected intensity $v_{k}\left(x\right)$ at time *k* using the detection probability $p_{D,k}$ and the clutter density $\kappa_{k}\left(z\right)$. The update is calculated by iterating over all measurements z of the measurement set $Z_{k}$ [11]:

(7) $$v_{k}\left(x\right)=\left(1-p_{D,k}\right)v_{k|k-1}\left(x\right)+\sum_{z\in Z_{k}}v_{D,k}\left(x;z\right)$$

with

(8) $$\begin{array}{cc} v_{D,k}\left(x;z\right) & =\sum_{j=1}^{J_{k|k-1}}w_{k}^{\left(j\right)}\left(z\right)N\left(x;m_{k|k}^{\left(j\right)}\left(z\right),P_{k|k}^{\left(j\right)}\right), \end{array}$$

(9) $$\begin{array}{cc} w_{k}^{\left(j\right)}\left(z\right) & =\frac{p_{D,k}w_{k|k-1}^{\left(j\right)}q_{k}^{\left(j\right)}\left(z\right)}{\kappa_{k}\left(z\right)+p_{D,k}\sum_{l=1}^{J_{k|k-1}}w_{k|k-1}^{\left(l\right)}q_{k}^{\left(l\right)}\left(z\right)}, \end{array}$$

(10) $$\begin{array}{cc} q_{k}^{\left(j\right)}\left(z\right) & =N\left(z;H_{k}m_{k|k-1}^{\left(j\right)},R_{k}+H_{k}P_{k|k-1}^{\left(j\right)}H_{k}^{T}\right), \end{array}$$

(11) $$\begin{array}{cc} m_{k|k}^{\left(j\right)}\left(z\right) & =m_{k|k-1}^{\left(j\right)}+K_{k}^{\left(j\right)}\left(z-H_{k}m_{k|k-1}^{\left(j\right)}\right), \end{array}$$

(12) $$\begin{array}{cc} P_{k|k}^{\left(j\right)} & =\left[I-K_{k}^{\left(j\right)}H_{k}\right]P_{k|k-1}^{\left(j\right)}, \end{array}$$

(13) $$\begin{array}{cc} K_{k}^{\left(j\right)} & =P_{k|k-1}^{\left(j\right)}H_{k}^{T}{\left(H_{k}P_{k|k-1}^{\left(j\right)}H_{k}^{T}+R_{k}\right)}^{-1}. \end{array}$$

Here, $p_{D,k}$ is the detection probability and $\kappa_{k}\left(z\right)$ is the clutter density, while updating of the Gaussian components is performed using the observation matrix $H_{k}$ and the observation noise covariance matrix $R_{k}$.

The equations presented here are the basis of the proposed tracking approach. However, various optimizations and adaptations are proposed around them in order to create an implementation that allows a fast and reliable application in real-world scenarios. These can be found in the following sections.

## 3. Proposed Multi-Sensor Multi-Object Tracking Approach

Multi-sensor multi-object tracking is considered here as estimating the number of all surrounding objects as well as their states over time while assigning track attributes, a confidence rating, and unique IDs to form trajectories. Therefore, the *i*-th tracked object $T_{k}^{\left(i\right)}$ of time step *k* is represented by a tuple $T_{k}^{\left(i\right)}=\left(\tau_{k}^{\left(i\right)},x_{k}^{\left(i\right)},c_{k}^{\left(i\right)},p_{k}^{\left(i\right)}\left(\exists \right)\right)$ including the track’s unique ID $\tau_{k}^{\left(i\right)}$, state $x_{k}^{\left(i\right)}$, classification $c_{k}^{\left(i\right)}$, and probability of existence $p_{k}^{\left(i\right)}\left(\exists \right)$. The classification $c_{k}^{\left(i\right)}$ is represented as a probability distribution or pseudo-probability distribution over all possible classes, which is necessary for fusion and estimation over time. The set of all *n* tracked objects is $T_{k}=\left\{T_{k}^{\left(1\right)},T_{k}^{\left(2\right)},\cdots ,T_{k}^{\left(n\right)}\right\}$.

Multiple independent sensors that may have different FoVs update the tracks with measurements $z_{k}$ and classification measurements $c_{z,k}$. The goal is to estimate the states and attributes of all existing tracks using the independent measurements provided by different sensors. Smart sensors with integrated object tracking capabilities might be used in real-world applications by treating the tracks of each time step as individual measurements.

### 3.1. Kalman Filter Approach

A Kalman filter-based approach is implemented here for comparison, as many current systems use this approach. In Kalman filter pipelines such as [3,4,5,6,8], a separate filter estimates the states of each tracked object. This is supported by a gating and data association approach that maps the incoming measurements of a sensor to the existing tracks. In this implementation, the chi-square test following ([25], p. 112) for gating and a global nearest neighbor data association scheme [26] based on the Mahalanobis distance is used. The distances between detections and tracks are calculated using the Mahalanobis distance $D_{M}$ with the predicted measurement ${\hat{z}}_{k|k-1}$ and the innovation covariance matrix $S_{k}$,

(14) $$D_{M}\left(z\right)=\sqrt{{\left[z_{k}-{\hat{z}}_{k|k-1}\right]}^{T}S_{k}^{-1}\left[z_{k}-{\hat{z}}_{k|k-1}\right]}$$

and the association is then solved using the Hungarian algorithm [27]. A track management scheme is responsible for adding new appearing tracks and removing disappearing tracks accordingly. In this work, the Bayesian formulation for existence probability estimation from [28] is used as a basis for the track management. Non-updated tracks are removed if the existence probability is below a certain threshold, while tracks are forwarded if the existence probability is above a minimum threshold.

The state vector of each track contains the two-dimensional position $p_{x}$ and $p_{y}$, speed $v_{x}$ and $v_{y}$, and acceleration $a_{x}$ and $a_{y}$, as well as the Oriented Bounding Box (OBB) orientation ϕ and OBB dimensions, consisting of length *l*, width *w*, and height *h*. The state $x_{k}$ vector is described as follows:

(15) $$x_{k}={\left[\begin{array}{cccccccccc} p_{x} & p_{y} & v_{x} & v_{y} & a_{x} & a_{y} & l & w & h & \phi \end{array}\right]}^{T}.$$

Similarly, the measurement vector $z_{k}$ consists of the position $p_{x}$ and $p_{y}$ as well as the dimensions *l*, *w*, and *h* and orientation ϕ, resulting in $z_{k}={\left[\begin{array}{cccccc} p_{x} & p_{y} & l & w & h & \phi \end{array}\right]}^{T}$. The system dynamics are described by a constant acceleration model with transition function *f* and time difference Δt, as the last time step k−1 is defined by

(16) $$x_{k|k-1}=f\left(x_{k-1|k-1},u_{k-1}\right)=\left[\begin{array}{c} p_{x}+\Delta tv_{x}+\frac{1}{2}\Delta t^{2}a_{x}+\Delta p_{x,u}\;\\ p_{y}+\Delta tv_{y}+\frac{1}{2}\Delta t^{2}a_{y}+\Delta p_{y,u}\;\\ v_{x}+\Delta ta_{x}\\ v_{y}+\Delta ta_{y}\\ a_{x}\\ a_{y}\\ l\\ w\\ h\\ \phi +\Delta \phi_{u} \end{array}\right]$$

with the control vector $u_{k-1}={\left[\begin{array}{cc} v_{ego} & \omega_{ego} \end{array}\right]}^{T}$ including the ego velocity $v_{ego}$ and the ego yaw rate $\omega_{ego}$, which results in

(17) $$\begin{array}{cc} \Delta p_{x,u} & =-v_{ego}\Delta t+\sin\left(\omega_{ego}\Delta t\right)\omega_{ego}\Delta tp_{x}+\cos\left(\omega_{ego}\Delta t\right)\omega_{ego}\Delta tp_{y}, \end{array}$$

(18) $$\begin{array}{cc} \Delta p_{x,u} & =-\cos\left(\omega_{ego}\Delta t\right)\omega_{ego}\Delta tp_{x}+\sin\omega_{ego}\Delta t\omega_{ego}\Delta tp_{y}, \end{array}$$

(19) $$\begin{array}{cc} \Delta \phi_{u} & =-\omega_{ego}\Delta t. \end{array}$$

The extended Kalman filter equations are used for the prediction.

### 3.2. Substituting the KF with the GM-PHD Filter

**Figure 1:**
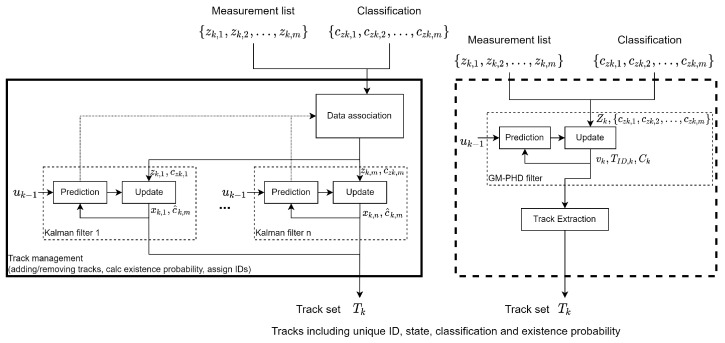
Comparison of KF and GM-PHD framework for automotive MOT application.

In order to replace a KF-based Multi-Object Tracking (MOT) framework with a GM-PHD-based one, the interfaces must be similar. The input of a KF framework is a list of detections, each containing a measurement vector $z_{k}$ and attributes, such as the classification $c_{z}$. Figure 1 shows an overview of the Kalman filter framework on the left-hand side and substitution by a GM-PHD framework on the right-hand side. With the KF, the states are estimated separately for each track. This means that the additional attributes, such as the classification, can also be calculated separately for each track. In contrast, the GM-PHD filter estimates the global PHD. The tracks are then extracted from the PHD in a separate step. Section 3.4 shows an approach to propagating additional attributes such as the classification alongside the PHD to provide this additional information for the extracted tracks.

### 3.3. Sensor-Based Parameter Models

#### 3.3.1. Detection Probability

**Figure 2:**
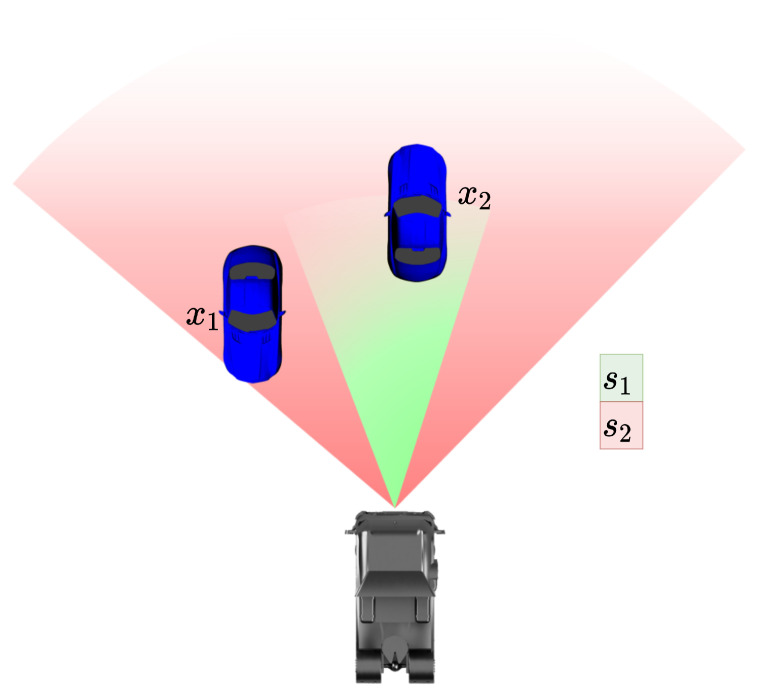
Example of two sensors $s_{1}$ and $s_{2}$ covering different FoVs and the states $x_{1}$ and $x_{2}$ of two tracked objects.

The original implementation of the GM-PHD filter [11] assumes a constant detection probability over the entire state space. However, this assumption is not the case with real sensor systems. For example, if a track with state $x_{1}$ is located outside the FoV of the sensor $s_{1}$, as shown in Figure 2, then the detection probability is 0; depending on the sensor technology, the detection probability will also change with environmental influences such as the weather or illumination conditions. For almost all sensors, there is a decrease in capability with increasing distance, which can be modeled mathematically.

The detection probability model is an important part of proper sensor fusion in the proposed framework; it strongly influences the appearance and disappearance, as it highly affects the weight calculation of the GM-PHD. In this framework, if a Gaussian component has a high detection probability and no corresponding measurement exists, then the weight will decrease; in turn, if a component has a very low detection probability and no corresponding measurement exists, then the weight is barely effected. Note that the detection probability model can also be applied to the existence probability estimation used for the KF framework.

We propose a detection probability model based on the FoV and the track’s distance. Therefore, a mathematical model $p_{Dm,k}^{\left(s_{p}\right)}\left(x\right)$ for the detection probability of track x is defined based on experimental measurements for each sensor $s_{p}$. Next to the FoV, which obviously sets limits to the detection probability, the distance is a key parameter. Due to the decreasing resolution at higher distances, the detection probability will decrease with the distance for all types of perception sensors. In addition, certain sensors, such as automotive radars, can have direction-dependent sensitivities and maximum detection ranges, which can result in more complex detection probability models. To ensure general applicability to different types of sensors, the proposed model is based only on the distance and a radial FoV.

Overall, the formula for the detection probability model $p_{Dm,k}^{\left(s_{p}\right)}\left(x\right)$ is as follows:

(20) $$\begin{array}{cc} p_{Dm,k}^{\left(s_{p}\right)}\left(x\right) & =p_{dist}^{\left(s_{p}\right)}\left(x\right)p_{FoV}^{\left(s_{p}\right)}\left(x\right),\\ p_{dist}^{\left(s_{p}\right)}\left(x\right) & =k_{2}^{\left(s_{p}\right)}d{\left(x\right)}^{2}+k_{1}^{\left(s_{p}\right)}d\left(x\right)+k_{0}^{\left(s_{p}\right)},\\ p_{FoV}^{\left(s_{p}\right)}\left(x\right) & =\left\{\begin{array}{cc} 0, & x\;\mathrm{out}\;\mathrm{FoV}\\ 1, & x\;\mathrm{in}\;\mathrm{FoV} \end{array}\right.. \end{array}$$

In this description, $p_{dist}^{\left(s_{p}\right)}\left(x\right)$ is the second-order polynomial describing the distance dependency, with $d\left(x\right)=\sqrt{p_{x}^{2}+p_{y}^{2}}$ being the distance of the track’s OBB center.

To verify this model and calculate the parameter values, the detection probabilities were experimentally determined for the “Car” class. Using the Point-RCNN [29] detector for Lidar and Yolo-Mono-3D [30] for monocular camera, the detection probability was calculated using the training data from the KITTI tracking dataset. For each time step, each of the ground truth tracks was compared with the respective measurements. If a measurement with an overlapping OBB was found for a ground truth track, it was counted as a true detection. Next, the percentage of detected tracks within the sensor FoV was calculated depending on the distance in order to obtain the detection probabilities.

**Figure 3:**
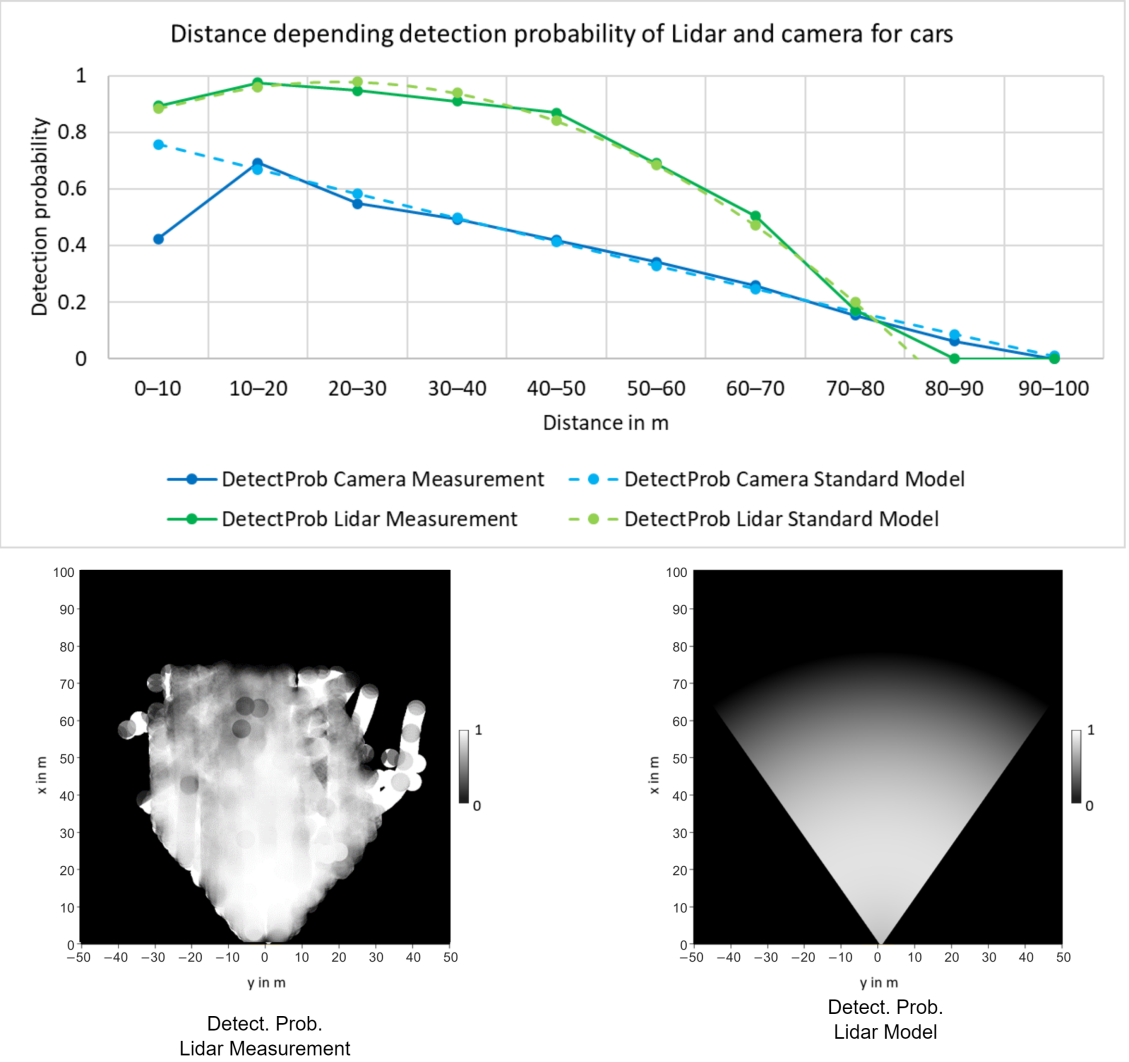
Detection probability distance model for “Car” class. On the top, the detection probability models of the camera and Lidar with respect to the distance; on the bottom, the detection probability measurements and model in BEV.

As shown on the top of Figure 3, the second-order polynomial used by the model can accurately represent the distance dependent detection probability for both the camera and the Lidar detector. The Bird’s Eye View (BEV) result of this polynomial model in combination with the sensor’s FoV is shown at the bottom of Figure 3 and compared to the measured result.

The GM-PHD implementation from [11] assumes the detection probability to be constant, but shows a closed-form update for a state-dependent detection probability provided as a mixture. However, this form results in numerous Gaussian components, which greatly increases the computational effort, as shown in Section 4.4. To reduce this, the constant assumption is violated and

(21) $$p_{D,k}=p_{Dm,k}^{\left(s_{p}\right)}\left(m_{k|k-1}^{\left(j\right)}\right)$$

is used instead. The experiments in Section 4.3 show that the proposed solution still works for real-world data. In [16], state-dependent detection probability was similarly used without a mixture model. However, their emphasis was on FoV and occlusion rather than on presenting a comprehensive model that encompasses distance dependency. Likewise, ref. [19] differentiated the detection probability depending on the object being inside or outside the FoV, and did not consider the distance dependency.

#### 3.3.2. Clutter Density

Similar to the detection probability, the clutter density is described as a mathematical model. In contrast to the detection probability, the GM-PHD standard filter equations do not include the assumption of a static value. Therefore, a mathematical model for the clutter density can be used directly.

The clutter density can be modeled similar to the detection probability. Therefore, the mathematical model for $\kappa_{k}^{\left(s_{p}\right)}\left(z\right)$ of sensor $s_{p}$ is experimentally defined based on the distance.

**Figure 4:**
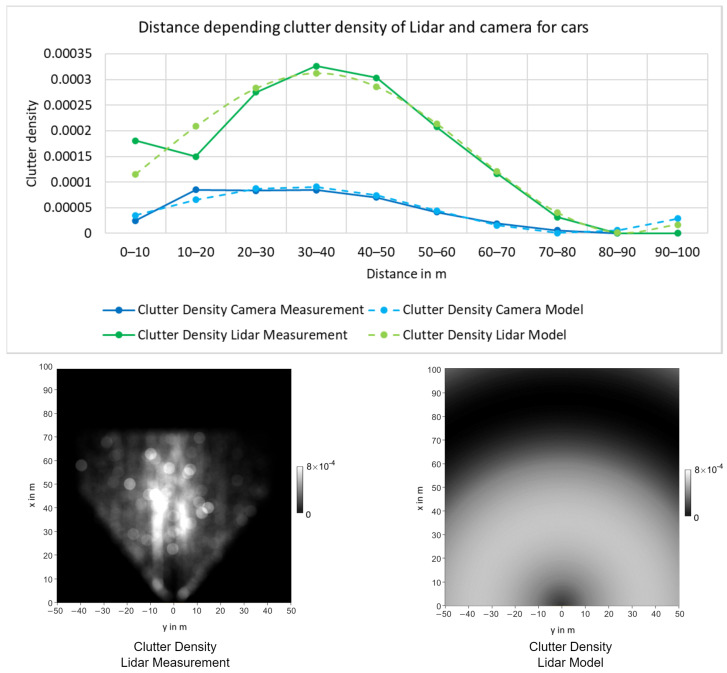
Clutter density distance model for "Car" class. On the top, clutter density models of the camera and Lidar with respect to the distance; on the bottom, the Lidar clutter density measurement and model in BEV.

The distance dependency was experimentally determined using the Point-RCNN [29] detector for Lidar and Yolo-Mono-3D [30] for monocular camera on the training data of the KITTI tracking dataset. Figure 4 shows the measured clutter density of the “Car” class for both the Lidar and the camera. As shown, the clutter density has a peak value for medium distances and appears to be lower for both near and far distances. This behavior can be modeled using a sinusoidal function, which leads to the approximations shown in Figure 4.

The overall mathematical description for the clutter density $\kappa_{k}^{\left(s_{p}\right)}\left(z\right)$ of measurement z is described by

(22) $$\kappa_{k}^{\left(s_{p}\right)}\left(z\right)=k_{0}\sin\left(k_{1}d\left(z\right)+k_{2}\right)+k_{0}.$$

### 3.4. Computational Steps for the GM-PHD Approach

This section describes the processing steps of the approach using the GM-PHD filter. These steps use the GM-PHD filter equations, and additionally propagate the tags for the unique ID and the classification over time. As described by [23], a unique identifier, or tag, $\tau_{k|k}$, is assigned to each Gaussian component at time step *k*, which together form a set

(23) $$\tau_{k|k}=\left\{\begin{array}{ccc} \tau_{k|k}^{\left(1\right)}, & \cdots , & \tau_{k|k}^{\left(J_{k|k}\right)} \end{array}\right\},$$

where $J_{k|k}$ is the number of Gaussian components at time step *k* and $\tau_{max}$ is the currently highest unique ID. To additionally track the classification of each Gaussian component, the respective classification set $C_{k|k}$ with

(24) $$C_{k|k}=\left\{\begin{array}{ccc} c_{k|k}^{\left(1\right)}, & \cdots , & c_{k|k}^{\left(J_{k|k}\right)} \end{array}\right\}$$

is introduced.

#### 3.4.1. Step 0: Initialization

For initialization, the number of Gaussian components is set to $J_{0}=0$ and the tags and classification sets are empty, with $\tau_{0}=\emptyset$ and $C_{0}=\emptyset$. After the one-time initialization, steps 1–5 are repeated.

#### 3.4.2. Step 1: Prediction

The prediction step predicts the PHD at the current time step using the constant acceleration model from Section 3.1 and the extended Kalman PHD equations from [11]. In addition, the survival probability $p_{S,k}$ is calculated for each time step using a base survival probability $p_{S,base}$ and the time difference from the last time step Δt, as follows:

(25) $$p_{S,k}=p_{S,base}^{\Delta t}.$$

The proposed implementation does not model the spawning process of Equation (2), which ignores crtain scenarios for simplicity and sets $v_{\beta ,k|k-1}\left(x\right)=0$. New objects can appear anywhere in the monitored space; however, as the computational effort should be kept low, we want to avoid creating a high number of random Gaussian component. Thus, adaptive birth models depending on the measurements are considered here, and a new adaptive approach is presented. In [31,32], the authors proposed adaptive birth processes which create new Gaussian components based on all measurements. However, this inevitably creates an overlap between birth components and already-tracked components in many areas. These overlaps do not truly represent the birth of new targets. Instead, Gaussian components should only be added in areas where no components are currently present. In this way, the influence of the newly added components on the update of the existing components is reduced, and both the total number of Gaussian components and the computational effort can be reduced while ensuring that the birth of new targets remains well-modeled.

In the presented approach, a birth measurement set $Z_{\gamma ,k-1}$ is generated in the previous update step k−1 that includes all measurements with a sum of generated weights $w_{sum,k-1}^{\left(j\right)}$ below a threshold $t_{w_{\gamma }}$:

(26) $$Z_{\gamma ,k-1}=\left\{z_{\gamma ,k-1}^{\left(j\right)}\in Z_{k-1}\;\forall \;w_{sum,k-1}^{\left(j\right)}<t_{w_{\gamma }}\right\}.$$

The threshold was found experimentally and set to $t_{w_{\gamma }}=0.01$. For the sum of weights, the measurement weights $q_{k-1}^{\left(l\right)}\left(z\right)$ from Equation (10) are used combined with the predicted weights $w_{k-1|k-2}$ from the previous time step:

(27) $$w_{sum,k-1}^{\left(j\right)}=\sum_{l=1}^{J_{k-1|k-2}}w_{k-1|k-2}^{\left(l\right)}q_{k-1}^{\left(l\right)}\left(z\right).$$

The sum of weights $w_{sum,k-1}^{\left(j\right)}$ indicates the influence of the *j*-th measurement on the PHD update. If the influence is very low, it is assumed that no Gaussian component of the intensity function is in close range to the measurement. Therefore, $Z_{\gamma ,k-1}$ is a subset of the measurement set $Z_{k-1}$ that only contains measurements in areas where no Gaussian components are present in the intensity function. Next, a Gaussian mixture ${\hat{\gamma }}_{k-1}$ is generated based on these measurements with $m_{\gamma ,k-1}^{\left(j\right)}={\left[\begin{array}{cccccccccc} p_{x}^{\left(j\right)} & p_{y}^{\left(j\right)} & 0 & 0 & 0 & 0 & l^{\left(j\right)} & w^{\left(j\right)} & h^{\left(j\right)} & \phi^{\left(j\right)} \end{array}\right]}^{T}$, $P_{\gamma ,k-1}^{\left(j\right)}=P_{0}$ with initial covariance $P_{0}$ and $w_{\gamma ,k-1}^{\left(j\right)}=w_{0}$ and with initial weight $w_{0}$. Then, ${\hat{\gamma }}_{k-1}$ is predicted to time step *k* using the GM-PHD prediction to form $\gamma_{k}$.

For each of the birth Gaussian components, a new birth tag is added along with the set of birth tags at the current time step, as proposed by [23]. Each tag is initialized with a new unique ID based on $\tau_{max}$, which is increased by $J_{\gamma_{k}}$ afterwards:

(28) $$\begin{array}{cc} \tau_{k|k-1} & =\tau_{k-1}\cup \left\{\begin{array}{ccc} \tau_{\gamma_{k}}^{\left(1\right)}, & \cdots , & \tau_{\gamma_{k}}^{\left(J_{\gamma_{k}}\right)} \end{array}\right\}, \end{array}$$

(29) $$\begin{array}{cc} \tau_{\gamma_{k}}^{\left(j\right)} & =\tau_{max}+j. \end{array}$$

Similarly, a classification probability vector is added for each Gaussian component. The initial classification is calculated by updating a uniform classification distribution $c_{U}$ with the measurement classification probability vector $c_{z_{\gamma ,k-1}}^{\left(j\right)}$. In principle, this can be done with any classification update function $f_{c}\left(c,c_{z}\right)$ capable of updating the classification in a meaningful way; however, this is not further described here. The classification set is accordingly extended with

(30) $$\begin{array}{cc} C_{k|k-1} & =C_{k-1}\cup \left\{\begin{array}{ccc} c_{k|k-1}^{\left(1\right)}, & \cdots , & c_{k|k-1}^{\left(J_{\gamma_{k}}\right)} \end{array}\right\}, \end{array}$$

(31) $$\begin{array}{cc} c_{k|k-1}^{\left(j\right)} & =f_{c}\left(c_{U},c_{z_{\gamma ,k-1}}^{\left(j\right)}\right). \end{array}$$

#### 3.4.3. Step 2: Update

The update step follows the overall procedure from [23], with adaptations for improved tracking results and computation speed in ADAS applications.

A gating step is introduced to improve the computation speed. The GM-PHD update step adds many Gaussian components due to the update of each prior Gaussian component with each measurement component. Many of these components have very low weights and would be pruned in the next step anyway. To reduce this number of low-weight components in advance, a gating process is introduced. Because gating in ADAS applications can be interpreted spatially, the gating is based solely on the position in terms of the X and Y coordinates. The minimum of the squared Mahalanobis distance $D_{M,pos}$ and the Euclidean distance $D_{Euclid,pos}$ defining the gate are calculated using the position difference $z_{p,zm}^{\left(i\right)}$ and the position covariance $P_{p}^{\left(i\right)}$, which only include the elements related to the X–Y position:

(32) $$\begin{array}{cc} z_{p,zm}^{\left(i\right)} & ={\left[\begin{array}{cc} \left(z_{1}-m_{k|k-1,1}^{\left(i\right)}\right) & \left(z_{2}-m_{k|k-1,2}^{\left(i\right)}\right) \end{array}\right]}^{T}, \end{array}$$

(33) $$\begin{array}{cc} P_{p}^{\left(i\right)} & =\left[\begin{array}{cc} P_{k|k-1,11}^{\left(i\right)} & P_{k|k-1,12}^{\left(i\right)}\\ P_{k|k-1,21}^{\left(i\right)} & P_{k|k-1,22}^{\left(i\right)} \end{array}\right]. \end{array}$$

The distances are then calculated with

(34) $$\begin{array}{cc} D_{M,pos}\left(z_{p,zm}^{\left(i\right)},P_{p}^{\left(i\right)}\right) & ={\left[z_{p,zm}^{\left(i\right)}\right]}^{T}{\left[P_{p}^{\left(i\right)}\right]}^{-1}\left[z_{p,zm}^{\left(i\right)}\right], \end{array}$$

(35) $$\begin{array}{cc} D_{Euclid,pos}\left(z_{p,zm}^{\left(i\right)}\right) & =\sqrt{\left[z_{p,zm}^{\left(i\right)}\right]{\left[z_{p,zm}^{\left(i\right)}\right]}^{T}}. \end{array}$$

Applying the gating based on these distances, Equation (8) changes to

(36) $$v_{D,k}\left(x;z\right)=\sum_{j\in V_{k}\left(z,\gamma_{DA}\right)}w_{k}^{\left(j\right)}\left(z\right)N\left(x;m_{k|k}^{\left(j\right)}\left(z\right),P_{k|k}^{\left(j\right)}\right),$$

where only components of the gating indices $V_{k}\left(z,\gamma_{DA}\right)$ are taken into account. For these gating indices, either the squared Mahalanobis distance or the Euclidean distance is below the gating threshold $\gamma_{DA}$:

(37) $$V_{k}\left(z,\gamma_{DA}\right)=\left\{i=1,\cdots ,J_{k}\;|\;\min\left(D_{M,p}{\left(z_{p,zm}^{\left(i\right)},P_{p}^{\left(i\right)}\right)}^{2},D_{Euclid,p}\left(z_{p,zm}^{\left(i\right)}\right)\right)\leq \gamma_{DA}\right\}.$$

The update step uses the sensor-based parameter models proposed in Section 3.3 for the detection probability and clutter density. Therefore, the following applies and is used in the update equations:

(38) $$\begin{array}{cc} p_{D,k} & =p_{Dm,k}^{\left(s_{p}\right)}\left(m_{k|k-1}^{\left(j\right)}\right), \end{array}$$

(39) $$\begin{array}{cc} \kappa_{k}\left(z\right) & =\kappa_{k}^{\left(s_{p}\right)}\left(z\right). \end{array}$$

Because new Gaussian components are generated during the update step, new tags and classifications are added to the tag set and classification set with *m* measurements, as follows:

(40) $$\begin{array}{cc} \tau_{k,u} & =\left\{\tau_{k|k-1}\cup \tau_{k|k-1}^{z_{1}}\cup \cdots \cup \tau_{k|k-1}^{z_{m}}\right\}, \end{array}$$

(41) $$\begin{array}{cc} \tau_{k|k-1}^{z} & =\left\{\tau_{j}\in \tau_{k|k-1}\;|\;j\in V_{k}\left(z,\gamma_{DA}\right)\right\}, \end{array}$$

(42) $$\begin{array}{cc} C_{k,u} & =\left\{C_{k|k-1}\cup C_{k,u}^{z_{1}}\cup \cdots \cup C_{k,u}^{z_{m}}\right\}, \end{array}$$

(43) $$\begin{array}{cc} C_{k,u}^{z} & =\left\{f_{c}\left(c_{k}^{\left(j\right)},c_{z_{\gamma ,k-1}}^{\left(j\right)}\right)\;|\;j\in V_{k}\left(z,\gamma_{DA}\right)\right\}. \end{array}$$

For each Gaussian component created by a measurement, a tag is added that has the same value as the tag of the updated component. Similarly, for each Gaussian component created by a measurement, the classification probability vector is updated by the measurements’ classification probability vector $c_{z_{\gamma ,k-1}}^{\left(j\right)}$ using the function $f_{c}\left(c,c_{z}\right)$.

#### 3.4.4. Step 3: Pruning

The pruning step from [23] is implemented without adaptations, which means that all components with low weights below a truncation threshold τ are eliminated. The classification set $C_{k,u}$ is pruned in a similar way to the tag set.

#### 3.4.5. Step 4: Merging

The merging procedure in general follows the one proposed in [23], and has the goal of merging Gaussian components that have a low distance based on a distance criterion. In contrast to [11,23], and similar to [33], this approach uses the Kullback-Leiber Divergence (KLD) $D_{KL}$ as the distance criterion, which is defined as follows:

(44) $$D_{KL}(N_{i}\left|\right|N_{j})=\frac{1}{2}\left(\mathrm{tr}\left(P_{j}^{-1}P_{i}\right)-k+{\left(m_{j}-m_{i}\right)}^{T}P_{j}^{-}1\left(m_{j}-m_{i}\right)+\ln\left(\frac{\det P_{j}}{\det P_{i}}\right)\right).$$

Here, *k* is the dimension of the covariance matrices $P_{i}$ and $P_{j}$. This distance is well-suited to being used as a criterion for whether two Gaussian components should be merged, as it provides an indication of the possible information loss.

When merging multiple components, the tag $\tau_{k}^{\left(i\right)}$ of the component with the highest weight $w_{k}^{\left(i\right)}$ is kept for the merged component. If components with the same tag still exist after the merging procedure, the component with the highest weight keeps the tag and all others are assigned a new one, similar to the proposal from [23].

The classification is merged similarly to the mean. Therefore, the merged classification distribution vector ${\tilde{c}}_{k}$ is calculated using the weighted average of all merged components *L*:

(45) $${\tilde{c}}_{k}=\frac{1}{{\tilde{w}}_{k}}\sum_{i\in L}w_{k}^{\left(i\right)}c_{k}^{\left(i\right)}.$$

#### 3.4.6. Step 5: Track Extraction

In the last step, the tracks are extracted from the PHD estimation. To be interpreted as a track, the weight of a Gaussian component has to exceed a weight threshold $w_{\min}$ creating the track set $T_{k}$:

(46) $$\begin{array}{c} T_{k}=\left\{\left(\tau_{k}^{\left(i\right)},m_{k}^{\left(i\right)},c_{k}^{\left(i\right)},{\tilde{p}}_{k}^{\left(i\right)}\left(\exists \right)\right)\;|\;w_{k}^{\left(i\right)}>w_{\min}\right\}. \end{array}$$

To provide an existence probability for further processing steps, the weight of the Gaussian component is interpreted as a pseudo-existence probability with ${\tilde{p}}_{k}^{\left(i\right)}\left(\exists \right)=\min\left(w_{k}^{\left(i\right)},1\right)$.

### 3.5. Track Confirmation Strategy

After the track extractions, the relevant information for ADAS functions, such as the unique IDs, states, classifications, and pseudo-existence probabilities, are available; however, the tracks may still be unstable. The existence and stability of the tracks is determined by the calculated weights of the Gaussian components and the propagated ID tags in the GM-PHD filter. However, there are situations in which these methods reach their limits:

- Short-term occlusion of objects, where the track may disappear and a new one with a new ID is subsequently created.
- Clutter tracks created due to random false detections.
- If one of several sensors systematically fails to detect a target, the weight of the track may be low even though the object is tracked over a long period.

**Figure 5:**
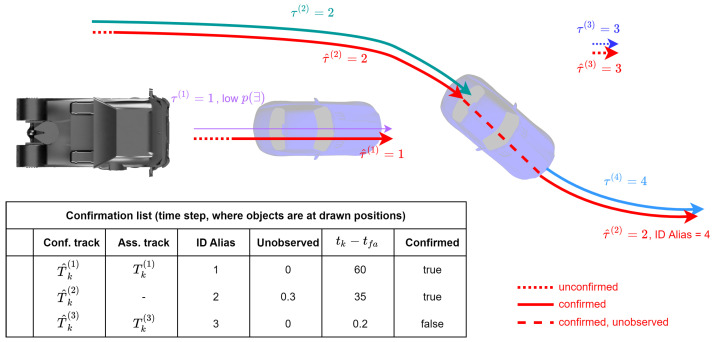
Example situation with track confirmation strategy. The trajectories of the confirmation list are drawn in red, while the trajectories of the track set have different colors. The confirmation list table shows example values at the time where the objects are at the cars’ positions.

A track confirmation strategy is applied to overcome these limitations and pass only those tracks confirmed based on several criteria to the ADAS function. This consists of a confirmation list that is updated after each update cycle based on the track set $T_{k}$. Each element in the confirmation list consists of a confirmation track ${\hat{T}}_{k}^{\left(i\right)}$, an ID alias, the unobserved time, the time of first appearance $t_{fa}$, and a confirmation flag, as shown in the example in Figure 5. The unobserved time is the time since the ID of the track was last updated. Figure 5 shows an example scenario with two cars around the ego vehicle. The arrows show the trajectories of the tracks provided by MOT. One car is directly in front of the ego vehicle, but the provided existence probability is low. The other car is overtaking and occluded for a short duration, during which the tracks are lost. In addition, there is a false positive track in the right top corner. This scenario represents several different errors that can possibly occur for a MOT system.

In each update cycle of the confirmation strategy, the tracks in the confirmation list should be updated. Here, the ID alias of the stored confirmation track is compared with the ID of the tracks in the track set. If no match is found, then the unobserved time is increased and the state is predicted using the motion model. In Figure 5, this is the case during occlusion of the overtaking car with ${\hat{\tau }}^{\left(2\right)}=2$. Otherwise, the unobserved time is set to 0.

Next, ID switches are searched for all tracks in the confirmation list that have not yet received an assignment from the update track set. If a track is found in the update track set for a non-updated track in the confirmation list for which the Euclidean distance of the location is below the threshold, then an ID change is assumed and the ID alias is set to the new ID, which is indicated in Figure 5 when the occluded car is tracked again. In this way, objects that have been lost for a short time, e.g., due to occlusion, can be found again while preserving their original unique ID. For all tracks in the update track set that have not yet been assigned to a track in the confirmation list, a new element is created in the confirmation list.

For all elements in the list, the next step checks whether they can be marked as confirmed. To be confirmed, one of the following criteria must be met:

- $p\left(\exists \right)>p_{\exists ,min}\wedge t_{k}-t_{fa}>t_{min}$
- $t_{k}-t_{fa}>t_{conf}$

In order to be confirmed, a track must either have an existence probability p(∃) greater than a threshold $p_{\exists ,min}$ and at the same time be tracked for a minimum duration $t_{min}$, or be tracked for a duration greater than $t_{conf}$ regardless of the existence probability. In Figure 5, the car with low existence probability is confirmed, as it has been tracked for a long duration, while the false positive track is not confirmed, and consequently is not forwarded to the ADAS functions. All tracks in the confirmation list are then checked for deletion using the unobserved time. If the unobserved time is above the threshold, they are removed from the list. The threshold for already-confirmed tracks is higher than the threshold for unconfirmed tracks.

This confirmation strategy can filter out some of the errors shown in Figure 5. In particular, situations where ID changes occur or tracks are lost for a short period of time can be resolved. In addition, it can reduce the amount of false positive tracks provided to the ADAS application. Therefore, the confirmation strategy can help to improve overall system performance. In our experiments, the confirmation strategy was applied to both the GM-PHD approach and the Kalman approach.

## 4. Results

The proposed approach was evaluated on both the KITTI dataset [22] and a custom dataset recorded using a truck with multiple sensors that had partially overlapping sensor FoVs. The experiments show a comparison between the proposed approach and the KF reference approach on both datasets. In addition, the influence of the parameter models is shown and the runtimes are analyzed.

### 4.1. Datasets

**Figure 6:**
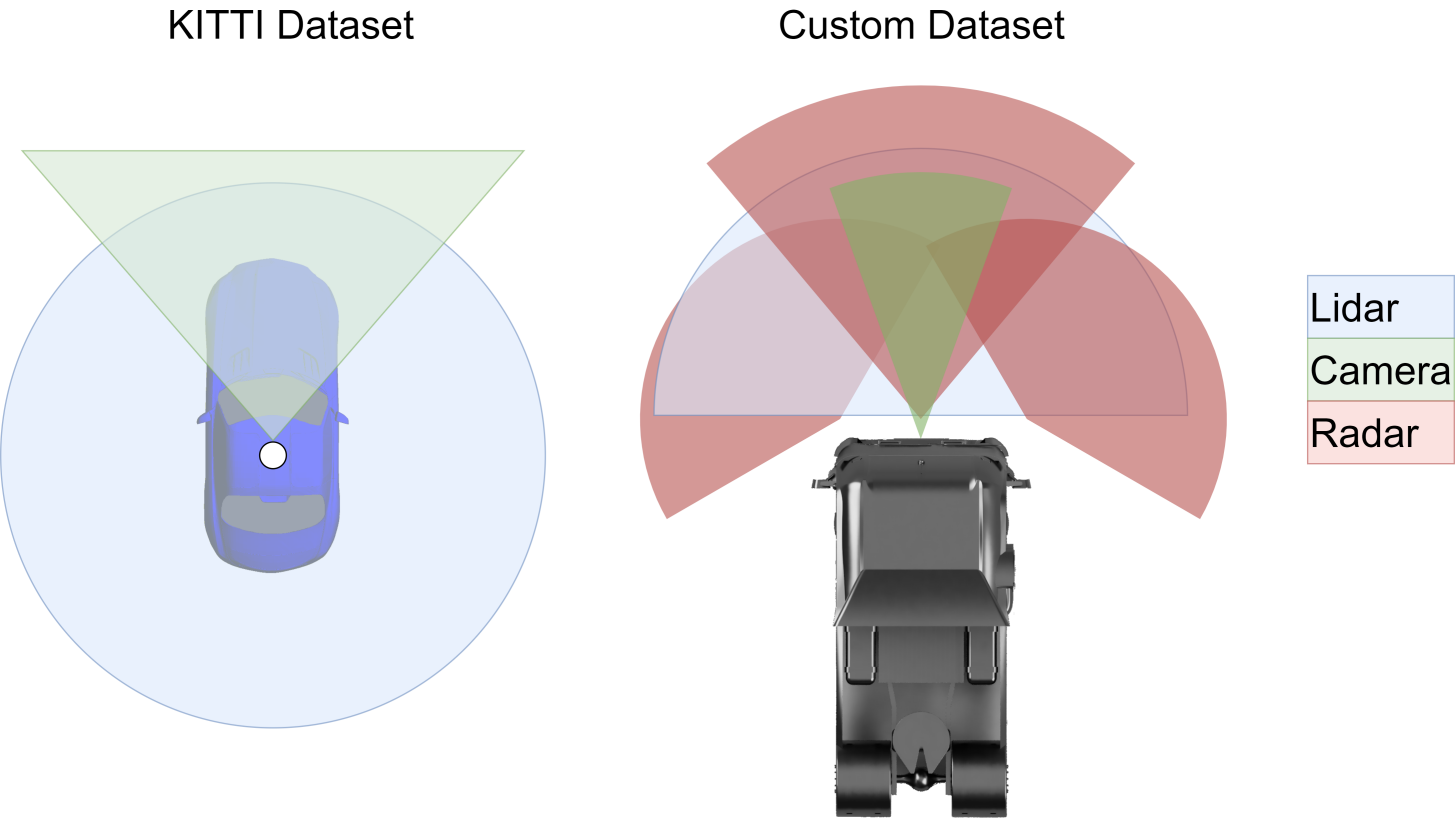
Comparison of dataset FoVs.

The sensor setup and sensor FoVs of both datasets are visualized in Figure 6. As shown in the figure, both datasets have a forward-facing camera, while the KITTI dataset uses a larger FoV. The custom setup relies more on radars, with two corner Short Range Radar (SRR) and a central Long Range Radar (LRR). The KITTI dataset is well suited for the comparisons in this paper, as it is well studied, widely used, and contains ground truth data. For the experiments, only objects of the “Car” type were examined, as they occur most frequently in the dataset and as such are the most meaningful.

A new custom dataset was created to evaluate the tracking and fusion algorithms on the basis of current generation sensors and validate the results from the KITTI dataset. This dataset included sensors comparable to truck series equipment with integrated object detection in order to prove the validity and performance of the presented approaches for use in current-generation vehicles. The Lidar sensor was only used to generate ground truth OBB data in these experiments, with the other sensors used for tracking. The objects provided by the camera and radar sensors are geometrically represented either as a point, as a point with estimated width, or as an L-shape, depending on the situation and distance. In order to create a common representation, the detections of all sensors were converted to point objects without spatial expansion; thus, the length, width, height, and orientation are ignored for the custom dataset.

The custom dataset is small compared to KITTI, and contains three scenes from different operational design domains (highway, rural, and urban). Due to its smaller size, the custom dataset is less meaningful; however, it can still serve for confirmation or questioning of the results on the KITTI dataset, and provides the opportunity to study the fusion of more than two sensors with different and partially non-overlapping FoVs.

### 4.2. Comparison of Kalman and GM-PHD Filters on Real-World Data

First, a comparison of the GM-PHD approach and the Kalman approach is carried out to show the general potential of the presented approach. Only the FoV-dependency of the detection probability is used, with the parameters otherwise modeled as constant, as the Kalman approach does not take such dependencies into account either. The parameters are optimized for the fused setup for both datasets, and are kept the same for single-sensor tracking. Manual optimization of the parameters was carried out with the goal of achieving optimal results for both GM-PHD and KF. In order to provide a direct comparison of the filter outputs and prevent the comparison from being primarily dependent on the final optimization, the parameters were kept the same for both. Slight improvements were still possible by fine-tuning the parameters depending on the filter; thus, the results for separately tuned parameters are provided for the custom dataset.

The comparison was first conducted using the KITTI dataset. The training dataset of the MOT benchmark was used to show multiple comparisons. The Higher Order Tracking Accuracy (HOTA) metric [34] was used as a performance measure. As the KITTI dataset only contains raw data, the Point-RCNN [29] detection approach was used to provide detections based on Lidar and Yolo-Mono-3D [30] was used to provide camera-based detections. Therefore, detections in the form of oriented bounding boxes suitable for processing are available. First, the performance of the Kalman and the GM-PHD approach is compared directly.

**Figure 7:**
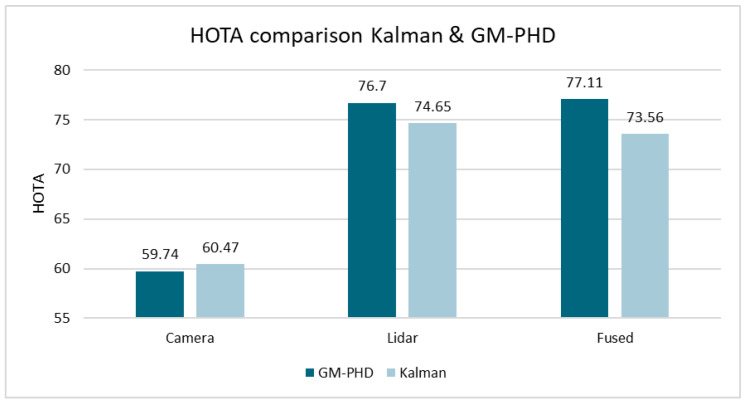
Comparison of HOTA results for Kalman and GM-PHD filters with different tracking setups on the KITTI dataset.

**Figure 8:**
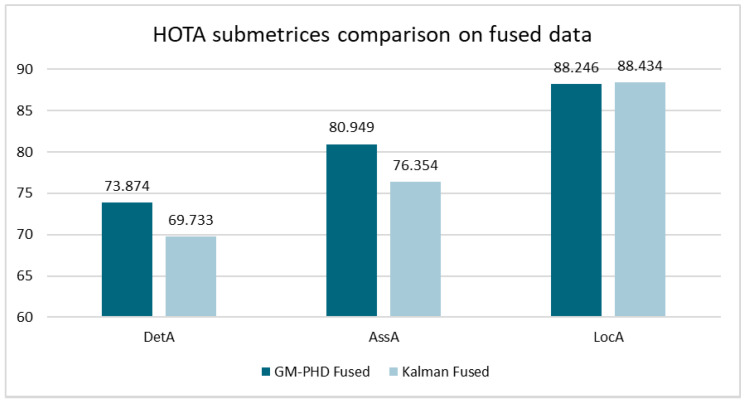
HOTA submetrices for fused tracking setup on the KITTI dataset.

Figure 7 shows the HOTA results of both approaches on the KITTI dataset. The comparison is shown when using only Lidar, only camera, and fused data. The GM-PHD approach clearly outperforms the Kalman approach for Lidar only, and even more so for fused data. When comparing the sub-metrics on the fused data in Figure 8, the differences are particularly evident for “DetA” and “AssA” [34], which are measures of detection accuracy and association accuracy. This indicates that the GM-PHD approach has a particular advantage when creating/deleting tracks and ensuring correct ID assignment.

**Figure 9:**
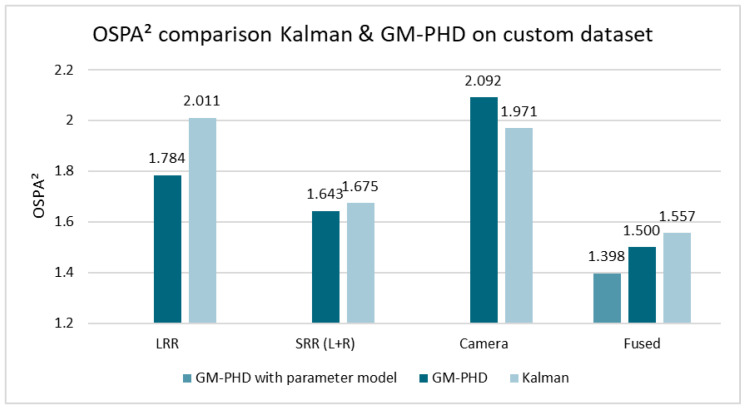
Comparison of OSPA^(2)^ error results for the Kalman and GM-PHD filters for different tracking setups on the custom dataset (smaller is better).

Both filters were then compared using the custom dataset. Here, the Second Order Optimal Sub-Pattern Assignment (OSPA^(2)^) metric [35] with cut-off c=2.5 and order p=1 was used, while the Euclidean distance was used as the base distance. Note that lower values of this metric represent a lower error. For a simplified comparison, the mean OSPA^(2)^ distance over all time steps of all three scenes was used. Figure 9 shows the results for the single-sensor tracking and fused setups. As shown, the GM-PHD filter achieves lower mean distances for all tracking setups except camera-only. In addition to the results from Figure 9, where the parameters were the same for the GM-PHD filter and the KF, the results for separately fine-tuned covariances are presented: the GM-PHD filter achieves an OSPA^(2)^ distance of 1.478, while the KF achieves 1.522 for the fused setup. While this performance difference is slightly decreased compared to the shared parameters, the GM-PHD approach still clearly outperforms the KF approach in this comparison.

Overall, the performance of the GM-PHD approach outperforms the Kalman approach in most tracking setups, and in particular for the fused data. The main advantages lie in its stable handling of appearing/disappearing tracks. For both datasets, the tracking performance in the camera-only setup is worse with the GM-PHD approach compared to the Kalman approach. This is probably due to the fact that the camera detections produce few clutter objects, but the position of the existing detections has a higher variance, which has a negative effect on the calculation of weights by the GM-PHD filter. In addition, the parameters are optimized for the fused setup and not fine-tuned for the single sensor tracking, which might have led to suboptimal results.

As shown by Figure 9, sensor-based parameter models can further reduce the OSPA^(2)^ distance, which is shown in detail in the following section.

### 4.3. Influence of Sensor-Based Parameter Models

**Figure 10:**
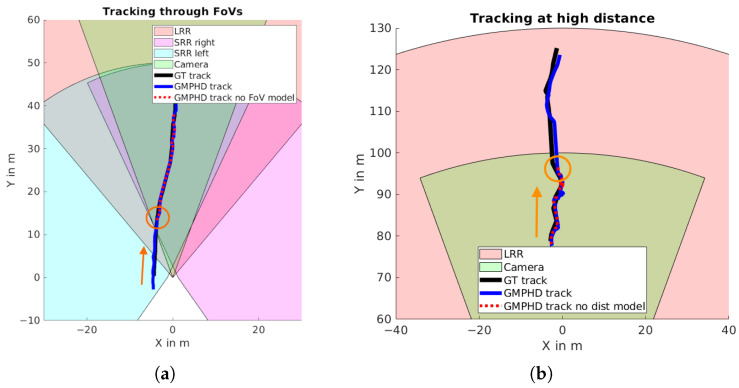
Example situations showing benefits of sensor-based parameter models. (**a**) An overtaking car moving in the direction of the orange arrow crosses multiple sensor FoVs. Stable tracking without the FoV model is reached at the overlap of all sensor FoVs. (**b**) Without the distance model for detection probability, the track of a car moving away in the direction of the orange arrow is lost at higher distances (the orange circle).

The sensor-based parameter models for detection probability and clutter density were evaluated with the GM-PHD approach. By applying the FoV to the detection probability, objects can be tracked through the FoVs of several sensors without applying additional rules. Figure 10a shows this on the custom dataset with an overtaking vehicle that is initially only in the FoV of the left SRR, then moves into the FoVs of all combined sensors. Without FoV modeling, stable tracking only occurs in the area of the combined view of all sensors (marked with an orange circle).

Because the detection capabilities of all sensors are quite different with respect to the distance, a similar effect occurs at higher distances, where some sensors are no longer capable of detecting an object while others remain able to detect it. Figure 10b shows a track from the custom dataset with a trajectory that is moving away from the ego vehicle. At a certain point, it is outside the capabilities of all sensors except the LRR; thanks to the distance-dependent model proposed here, it can still be tracked, while the track is lost when applying constant parameters (marked with an orange circle).

In addition to these detailed examples, a comparison of the performance with and without the sensor-based models was carried out for the tracking setups with sensor fusion. Because a comparison is not possible without modeling the FoV for the detection probability, this was applied to all scenarios, while the distance dependency was activated/deactivated depending on the test. Table 1 shows the results on the KITTI dataset and on the custom dataset. For both datasets, improvements can be achieved using the detection probability and the clutter density parameter model. However, the improvements on the KITTI dataset are rather small, while the improvements on the custom dataset show more potential. Here, the differences in the detection capabilities between the sensors is high in certain areas of the observed space and therefore needs to be modeled accordingly, which explains the higher improvements. In addition, the clutter density model shows less improvement for both datasets compared to the detection probability model. However, this is no limitation in the usage of the models, since improvements can be achieved in all situations, although they are small for some scenarios.

### 4.4. Runtime Evaluation

The runtime of the presented GM-PHD approach was compared with the Kalman approach on the KITTI dataset and on the custom dataset with the Lidar-only setup. In addition, a variant without the gating in the update step was compared, as well as a variant where the detection probability was calculated using a mixture with seven components, as described in [11]. Table 2 shows the HOTA results and the average runtime for one cycle. All approaches were implemented in C++ and tested on an Intel Core I7-9700K CPU.

The runtime can be greatly reduced without significantly affecting the HOTA results by the introduction of gating. The original variant with mixture implementation for the detection probability is considerably slower. Thanks to the presented optimizations, the runtime is only 2.5 times that of the Kalman filter, which is not possible with other GM-PHD approaches. This means that the approach presented here is expected to work in embedded devices.

In general, fast reactions are as important as accuracy for ADAS functions. If an emergency function needs to pursue the absolute minimum reaction times, KF might be the preferable choice. In real systems, it is necessary to calculate the updates for all sensors with cycle times of 10–20 Hz. If only limited computing power is available and these cycles cannot be achieved with the GM-PHD filter, then KF needs to be used. However, as KF has been used in sensor fusion systems for years and the available computing power continues to increase, experiments have shown that an update cycle of the GM-PHD filter can be calculated within the range of a millisecond; it can be assumed that this constraint can also be met by the GM-PHD filter in most systems nowadays. In these cases, the improved accuracy of the GM-PHD will benefit the system.

## 5. Conclusions

This paper shows a GM-PHD MOT approach that uses optimizations to enable easy application in real-time automotive systems. It supports multiple sensors with different FoVs and sensing capabilities by embracing sensor-based parameter models. Furthermore, the proposed approach introduces the possibility of propagating additional track properties, such as the classification, with the GM-PHD filter through time, thereby enhancing the filter’s versatility. The proposed approach is able to achieve low runtimes while exceeding a Kalman-based MOT in terms of tracking performance, and can handle the appearance and disappearance of objects as well as the data association problem implicitly due to its RFS properties. The proposed GM-PHD approach achieved an OSPA^(2)^ error of 1.40 on the custom dataset, compared to 1.56 for the KF approach; therefore, this paper shows that the proposed GM-PHD filter approach is very well suited as a substitute for KF in automotive MOT and fusion systems. This allows existing systems to be improved in the short term.

## Figures and Tables

**Table 1:** Results of sensor-based parameter models using fused tracking setup on both datasets.

	KITTI Dataset (HOTA)	Custom Dataset (OSPA^(2)^)
Constant model	77.11	1.50
$p_{d}$ with dist model	77.26	1.42
κ with dist model	77.15	1.48
$p_{d}$ and κ with dist model	77.34	1.40

**Table 2:** Runtime comparison of multiple test scenarios.

	HOTA	Runtime in ms
Kalman approach	74.65	0.285
GM-PHD as proposed	76.86	0.715
GM-PHD without gating	76.84	1.629
GM-PHD without gating and with mixture $p_{d}$	75.81	15.015

## Data Availability

Some or all data that support the findings of this study are available from the corresponding author upon reasonable request.

## Abbreviations

The following abbreviations are used in this manuscript:

ADAS	Advanced Driver Assistance Systems
BEV	Bird’s-Eye View
FoV	Field of View
GM-PHD	Gaussian Mixture Probability Hypothesis Density
HOTA	Higher-Order Tracking Accuracy
KF	Kalman Filter
KLD	Kullback–Leibler Divergence
LRR	Long-Range Radar
MOT	Multi-Object Tracking
OBB	Oriented Bounding Box
OSPA^(2)^	Second-Order Optimal Sub-Pattern Assignment
PHD	Probability Hypothesis Density
RFS	Random Finite Set
SRR	Short-Range Radar

## References

[1] Zaitouny A., Stemler T., Algar S.D. (2019). Optimal Shadowing Filter for a Positioning and Tracking Methodology with Limited Information. Sensors.

[2] Gustafsson F., Gunnarsson F., Bergman N., Forssell U., Jansson J., Karlsson R., Nordlund P.J. (2002). Particle filters for positioning, navigation, and tracking. IEEE Trans. Signal Process..

[3] Kampker A., Sefati M., Rachman A.S.A., Kreisköther K.D., Campoy P. Towards Multi-Object Detection and Tracking in Urban Scenario under Uncertainties. Proceedings of the 4th International Conference on Vehicle Technology and Intelligent Transport Systems (VEHITS 2018).

[4] Wu H., Han W., Wen C., Li X., Wang C. (2022). 3D Multi-Object Tracking in Point Clouds Based on Prediction Confidence-Guided Data Association. IEEE Trans. Intell. Transp. Syst..

[5] Mobus R., Kolbe U. Multi-target multi-object tracking, sensor fusion of radar and infrared. Proceedings of the IEEE Intelligent Vehicles Symposium.

[6] Chiu H.K., Prioletti A., Li J., Bohg J. (2020). Probabilistic 3D Multi-Object Tracking for Autonomous Driving. arXiv.

[7] Himmelsbach M., Luettel T., Wuensche H.J. Real-time object classification in 3D point clouds using point feature histograms. Proceedings of the 2009 IEEE/RSJ International Conference on Intelligent Robots and Systems.

[8] Schueler K., Weiherer T., Bouzouraa E., Hofmann U. 360 Degree multi sensor fusion for static and dynamic obstacles. Proceedings of the 2012 IEEE Intelligent Vehicles Symposium.

[9] Kalman R.E. (1960). A New Approach to Linear Filtering and Prediction Problems. J. Basic Eng..

[10] Mahler R.P.S. (2003). Multitarget Bayes filtering via first-order multitarget moments. IEEE Trans. Aerosp. Electron. Syst..

[11] Vo B.N., Ma W.K. (2006). The Gaussian Mixture Probability Hypothesis Density Filter. IEEE Trans. Signal Process..

[12] Liu L., Ji H., Zhang W., Liao G. (2019). Multi-Sensor Multi-Target Tracking Using Probability Hypothesis Density Filter. IEEE Access.

[13] Mahler R.P.S. Approximate multisensor CPHD and PHD filters. Proceedings of the 2010 13th International Conference on Information Fusion.

[14] Liu L., Ji H., Fan Z. (2016). A cardinality modified product multi-sensor PHD. Inf. Fusion.

[15] Li B., Ouyang W., Sheng L., Zeng X., Wang X. GS3D: An Efficient 3D Object Detection Framework for Autonomous Driving. Proceedings of the 2019 IEEE/CVF Conference on Computer Vision and Pattern Recognition (CVPR).

[16] Vasic M., Martinoli A. A Collaborative Sensor Fusion Algorithm for Multi-object Tracking Using a Gaussian Mixture Probability Hypothesis Density Filter. Proceedings of the 2015 IEEE 18th International Conference on Intelligent Transportation Systems.

[17] Li G., Yi W., Jiang M., Kong L. Distributed fusion with PHD filter for multi-target tracking in asynchronous radar system. Proceedings of the 2017 IEEE Radar Conference (RadarConf).

[18] Li G., Yi W., Li S., Wang B., Kong L. (2019). Asynchronous multi-rate multi-sensor fusion based on random finite set. Signal Process..

[19] Lindenmaier L., Aradi S., Becsi T., Toro O., Gaspar P. (2022). GM-PHD Filter Based Sensor Data Fusion for Automotive Frontal Perception System. IEEE Trans. Veh. Technol..

[20] Törő O., Bécsi T., Gáspár P. (2021). PHD Filter for Object Tracking in Road Traffic Applications Considering Varying Detectability. Sensors.

[21] Chen X., Li Y., Li Y., Yu J. (2018). PHD and CPHD Algorithms Based on a Novel Detection Probability Applied in an Active Sonar Tracking System. Appl. Sci..

[22] Geiger A., Lenz P., Urtasun R. Are we ready for autonomous driving? The KITTI vision benchmark suite. Proceedings of the 2012 IEEE Conference on Computer Vision and Pattern Recognition.

[23] Clark D., Panta K., Vo B.N. The GM-PHD Filter Multiple Target Tracker. Proceedings of the 2006 9th International Conference on Information Fusion.

[24] Panta K., Vo B.N., Clark D.E. An Efficient Track Management Scheme for the Gaussian-Mixture Probability Hypothesis Density Tracker. Proceedings of the 2006 Fourth International Conference on Intelligent Sensing and Information Processing.

[25] Challa S., Morelande M.R., Musicki D., Evans R.J. (2011). Fundamentals of Object Tracking.

[26] Konstantinova P., Udvarev A., Semerdjiev T., Rachev B., Smrikarov A. (2003). A study of a target tracking algorithm using global nearest neighbor approach. Proceedings of the 4th International Conference Conference on Computer Systems and Technologies e-Learning–CompSysTech’03.

[27] Kuhn H.W. (1955). The Hungarian method for the assignment problem. Nav. Res. Logist. Q..

[28] Aeberhard M. (2017). Object-Level Fusion for Surround Environment Perception in Automated Driving Applications. Ph.D. Dissertation.

[29] Shi S., Wang X., Li H. PointRCNN: 3D Object Proposal Generation and Detection From Point Cloud. Proceedings of the 2019 IEEE/CVF Conference on Computer Vision and Pattern Recognition (CVPR).

[30] Liu Y., Wang L., Liu M. YOLOStereo3D: A Step Back to 2D for Efficient Stereo 3D Detection. Proceedings of the 2021 IEEE International Conference on Robotics and Automation (ICRA).

[31] Houssineau J., Laneuville D. PHD filter with diffuse spatial prior on the birth process with applications to GM-PHD filter. Proceedings of the 2010 13th International Conference on Information Fusion.

[32] Ristic B. (2013). Particle Filters for Random Set Models.

[33] Granström K., Orguner U. On the reduction of Gaussian inverse Wishart mixtures. Proceedings of the 2012 15th International Conference on Information Fusion.

[34] Luiten J., Osep A., Dendorfer P., Torr P., Geiger A., Leal-Taixé L., Leibe B. (2021). HOTA: A Higher Order Metric for Evaluating Multi-object Tracking. Int. J. Comput. Vis..

[35] Beard M., Vo B.T., Vo B.N. (2020). A Solution for Large-Scale Multi-Object Tracking. IEEE Trans. Signal Process..

